# Structural Characteristics of the Lens in Presenile Cataract

**DOI:** 10.3389/fmed.2021.802275

**Published:** 2021-12-22

**Authors:** Sofija Andjelic, Kazimir Drašlar, Anastazija Hvala, Marko Hawlina

**Affiliations:** ^1^Eye Hospital, University Medical Centre, Ljubljana, Slovenia; ^2^Department of Biology, Biotechnical Faculty, University of Ljubljana, Ljubljana, Slovenia; ^3^Department of Pathology, Medical Faculty, University of Ljubljana, Ljubljana, Slovenia

**Keywords:** lens epithelial cells, presenile cataract, lens epithelium, cell morphology, scanning electron microscopy, transmission electron microscopy

## Abstract

The purpose of this work is to examine the structure of the anterior lens epithelial cells (aLECs) of presenile idiopathic cortical cataract to investigate the possible structural reasons for its development. The anterior lens capsules (aLCs: basement membrane and associated lens epithelial cells) were obtained from routine uneventful cataract surgery of 5 presenile cataract patients (16 and 41 years old women and 29, 39, and 45 years old men). None of the patients had family history of cataract, medication, or trauma and they were otherwise healthy. In addition, the patients did not have any other abnormal features in the ocular status except cataract. The aLCs were prepared for scanning electron microscopy (SEM) and transmission electron microscopy (TEM). The most prominent abnormal features observed by SEM for all 5 studied presenile cataract patients were the changes of the aLECs structure with the dents, the selective concavity of some LECs, at their apical side centrally toward the nucleus. In addition, TEM showed the thinning of the lens epithelium with the segmentally concave cells and the compressed and elongated nuclei. Abnormal and distinguishable structural features were observed in the anterior lens epithelium aLECs in all 5 patients with presenile cataract. Disturbed structure of aLECs, regularly present in presenile cataract type is shown that might be associated with water accumulation in the presenile idiopathic cortical cataract lens.

## Introduction

Presenile cataracts, such as juvenile cataracts, are rare. A presenile cataract is considered as a cataract found in a person under 45 years of age (1). Juvenile cataracts have an onset within the first decade of life (2). Juvenile and presenile cataracts can range from mild and benign to advance and sight-threatening and usually have a distinct structure. Presenile cataracts may have a hereditary cause, or can result from trauma or chromosomal, endocrine, metabolic, or systemic disorders (3). Yet, a sizeable percentage of presenile cataracts is of unknown cause. Presenile cataracts can be sporadic or familial. The mode of its inheritance can be dominant—principally in isolated forms or recessive—typical in syndromic forms (4). Among inherited non-syndromic cataract phenotypes, cataracts affecting the lens nucleus are common while cataracts limited to the lens cortex are rare (5). The lens epithelium structure in idiopathic cortical presenile cataract to the best of our knowledge was not studied up to now.

The lens epithelium is the first physical and biological barrier of the lens. It is metabolically the most active part, thus maintaining the physiological health of the lens (6). The lens epithelium is located in the anterior of the lens between the basement membrane and the lens fiber cells and is a cuboidal epithelium made from anterior lens epithelial cells (aLECs) (7). These cells have large-indented nuclei with two nucleoli and numerous pores (8). As the lens ages, aLECs become more flattened (8). In our previous studies, we have provided detailed evidence about the structural organization of the aLECs. With each of the three complementary techniques used, scanning electron microscopy (SEM), transmission electron microscopy (TEM), and confocal microscopy, we have shown that the apical surface of lens epithelial cells, oriented toward the fiber cells is smooth while at the basal surface, at the border with the lens capsule, the extensions and the entanglements of the cytoplasmic membrane of the lens epithelial cells are present (9). We have previously studied, by the complementary use of SEM and TEM, the structural features of the anterior lens epithelium in senile intumescent white cataract (10) and in retinitis pigmentosa (11). In senile intumescent cataracts' lens epithelium swollen cells, spherical formations and degraded cells were observed. In retinitis pigmentosa, holes and the degradation of the epithelium were observed with the dimensions from <1 μm to more than 50 μm.

In this work, the purpose was to study the structural features of the anterior lens epithelium in 1 case of juvenile and 4 cases of idiopathic presenile cortical cataract to investigate the possible structural reasons for its development.

## Materials and Methods

### Ethics Statement

The research followed the tenets of the Declaration of Helsinki. The study was approved by the National Medical Ethics Committee of the Republic of Slovenia and all patients signed informed consent before the operation.

### Patients

The aLCs were obtained by capsulorhexis from routine uneventful cataract surgery of 5 patients with presenile cataract (16 and 41 years old women and 29, 39, and 45 years old men) performed at the Eye Hospital, University Medical Centre (UMC), Ljubljana, Slovenia by the same surgeon (M.H.). None of the patients had a family history of cataract, medication, or trauma and they were otherwise healthy. None had any other abnormal features in the ocular status except cataract. In all five patients, the cataract was of cortical subcapsular type while in one, the cataract was of cortical intumescent type. All the patients were uneventfully operated in topical anesthesia. In three patients, preoperative examination included imaging with anterior segment optical coherence tomography (OCT) (Heidelberg Engineering Spectralis), for the other two, this was unavailable. The first patient was 16 years old female with juvenile cataract that caused gradual loss of vision with corrected visual acuity of 0.3 on the right and 0.6 on the left eye. The second patient was 41 years old female. On presentation, her corrected visual acuity was 0.5 on the right eye and 0.1 on the left eye. Left eye was operated and the posterior capsule was found to be partly fibrosed as well but did not significantly affect postoperative vision. The third patient was 29 years old male with corrected visual acuity on the right eye of 0.1 and 0.7 on the left eye. Right eye was operated and postoperative visual acuity was 0.7 due to fibrosis of the posterior capsule, which was later opened by YAG laser to gain distance visual acuity of 1.0. The fourth patient was 39 years old male with corrected preoperative visual acuity on the right eye of hand movement and 1.0 on the left eye. Right eye was operated and postoperative visual acuity was 1.0. The fifth patient was 45 years old male with corrected visual acuity on the right eye of 0.05 and 1.0 on the left eye. Right eye was operated and postoperative visual acuity was normal. In general, none of the patients had amblyopia or reduced visual acuity due to other ocular pathology.

### Tissue Preparation

The anterior capsules of one eye operated in five patients were studied. The 5–5.5 mm circles of the central aLC were carefully removed by continuous curvilinear capsulorhexis with forceps and cut in half. Each specimen was examined in detail both by SEM and TEM. One-half was immediately prepared for SEM performed at Biotechnical Faculty. A washing step was applied to the specimens with sodium cacodylate buffer 0.1 M, pH 7.2. Then, the specimens were double fixed: first, by 1% glutaraldehyde and 0.5% formaldehyde in 0.1 M cacodylate buffer, pH 7.2 for 2 h (25% glutaraldehyde EM grade; SPI and formalin were obtained from paraformaldehyde, Sigma); second, by 1% OsO_4_ in cacodylate buffer for 45 min. Dehydration of the aLC tissue was performed in an ethanol cascade. For drying of specimens, critical point drying (CPD, Balzers CPD 030 Critical Point Dryer) procedure with CO_2_ was applied. Dried specimens were glued by carbon adhesive discs to specimen stubs, then, Pt sputtered (Bal-Tec SCD 050 Sputter Coater) and examined in a field emission scanning electron microscope (FESEM, 7500 F, JEOL, Tokyo, Japan). The other half of the aLC was prepared for TEM at the Institute of Pathology. For TEM, specimens were immediately fixed in a solution of neutral buffered 10% formaldehyde and postfixated in 2% OsO_4_. All capsules were then dehydrated in increasing concentrations of ethanol and embedded in Epon 812. Semi-thin sections (1 μm) were made in the central part perpendicular to the capsule plane, stained with Azur II, in selected cases, they were also stained with Jones methenamine silver (JMS) and analyzed by using light microscopy. Ultrathin sections (30–50 nm) were stained with uranyl-acetate and lead-citrate and examined by a JEOL 1200 Ex II transmission electron microscope. Each specimen was examined in detail both by the SEM and the TEM.

## Results

**Figure 1:**
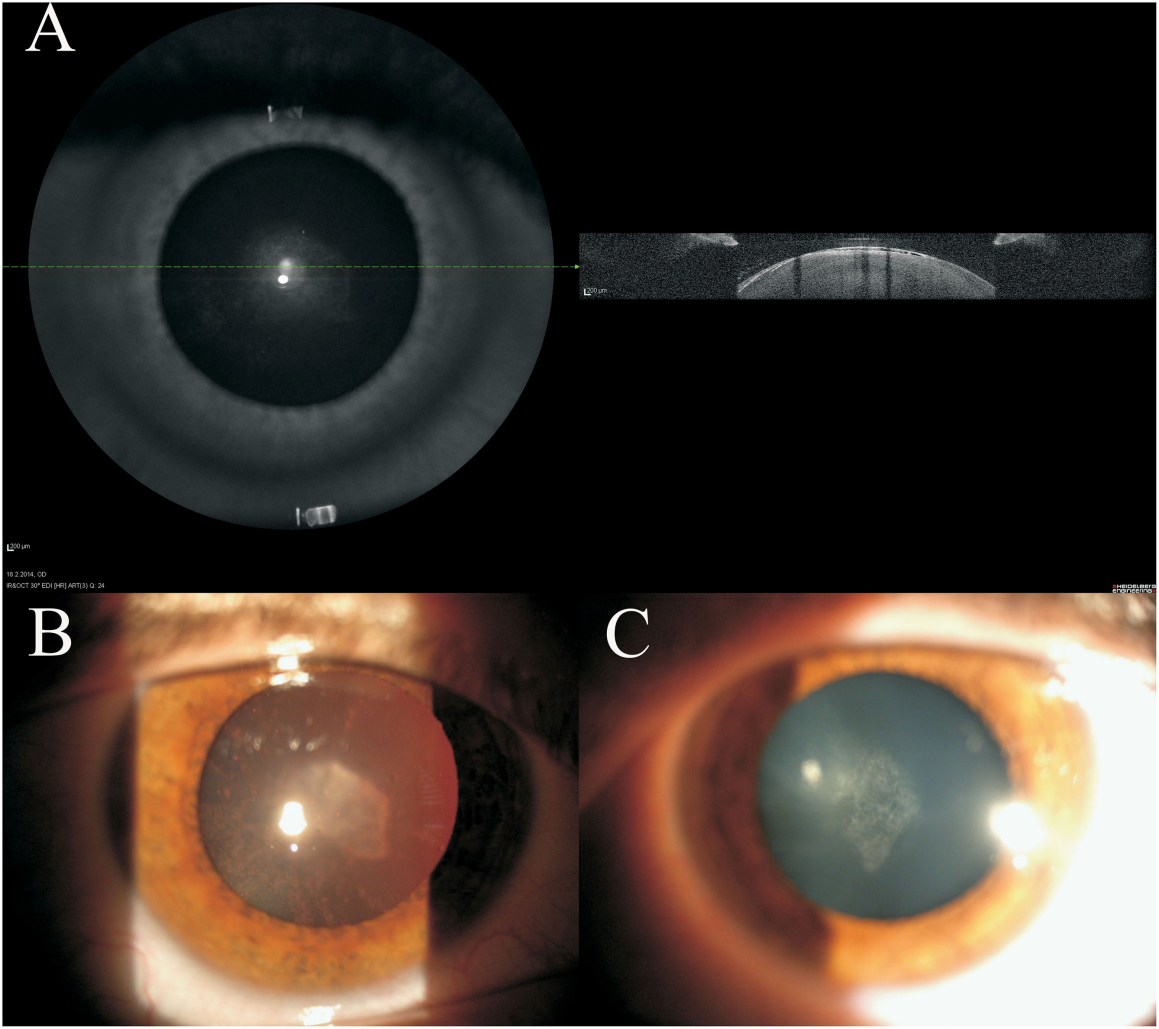
Clinical imaging of 16 years old female juvenile cataract patient's lens before cataract surgery. Spectralis optical coherence tomography (OCT) **(A)** and the slit lamp **(B,C)** images are shown. On the anterior part of the lens subcapsular opacities are visible.

Detailed photograph and OCT imaging of the lens were available for four patients: images of the 16-year-old female patient are shown in Figure 1, while in the supporting information Supplementary Figures S1, S2 images are available for the 39 and 45 years old male patients. In all three patients, subcapsular opacities and vacuoles between the lens capsule and cortex can be seen (Figure 1; Supplementary Figures S1, S2). The subcapsular changes in the opacity are visible on the anterior part of the lens as shown by the slit lamp (Figures 1B,C; Supplementary Figures S1B,C, S2B,C) and by the high-resolution Spectralis OCT images (Figure 1A; Supplementary Figures S1A, S2A). As shown on high-resolution Spectralis OCT images, vacuolation can be seen between the lens capsule and cortex (Figure 1A; Supplementary Figures S1A, S2A).

The following figures represent the results of the SEM and TEM study of the anterior lens epithelium of each patient. The most prominent abnormal features observed by SEM of all patients were the changes of the aLECs structure with the dents on their apical side centrally toward the nucleus.

### Patient 1 (16 Years Old Female)

**Figure 2:**
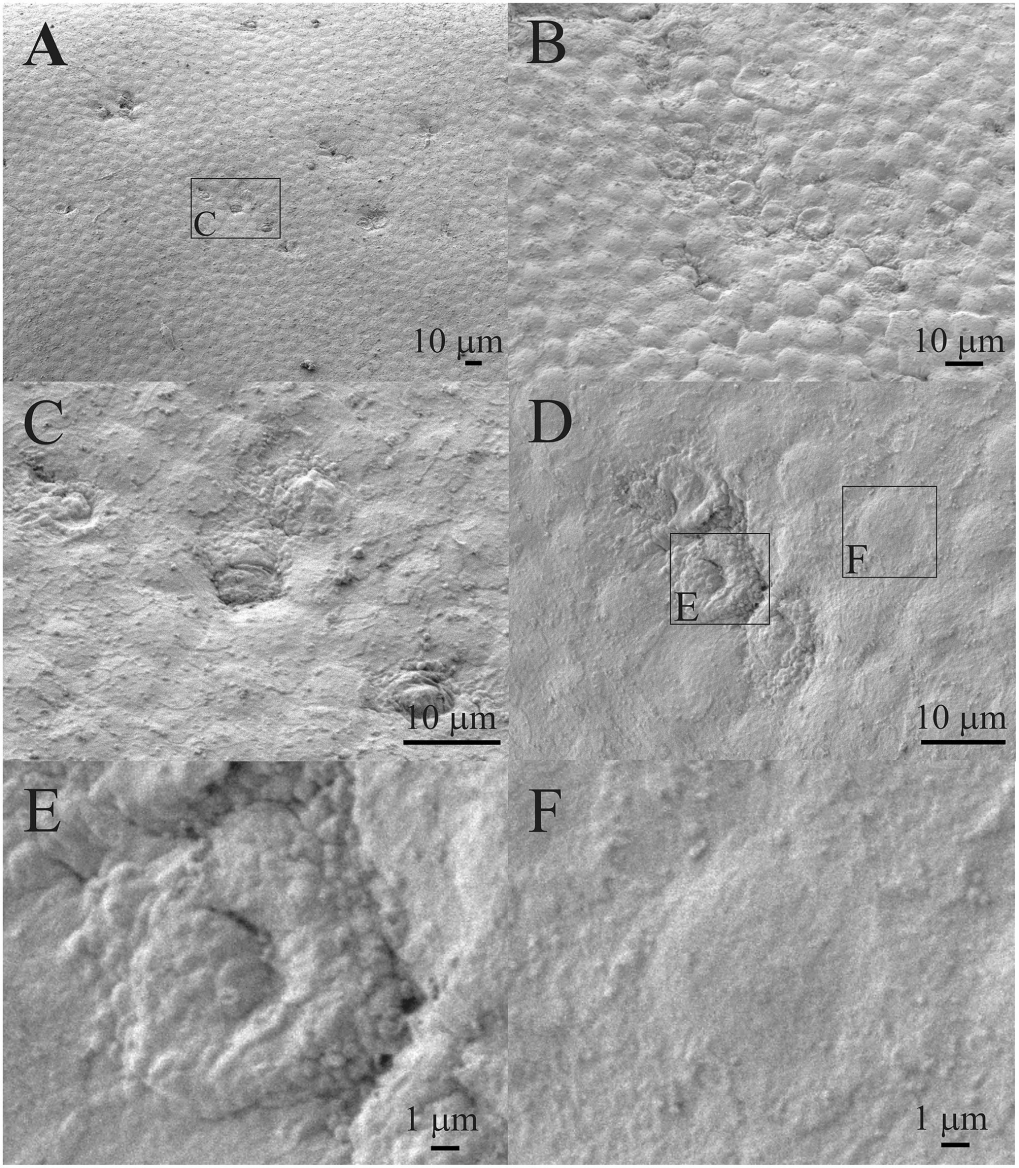
Scanning electron microscopy (SEM) of the 16 years old female juvenile cataract patient's anterior lens capsule (aLC). SEM is showing damaged individual anterior lens epithelial cells (aLECs) **(A,C)** or regions of lens epithelium **(B,D)**, with the dents on the apical side centrally toward the nucleus in the damaged aLECs **(D,E)**, and not the healthy aLECs **(D,F)**. The squares represent the regions that are enlarged and are labeled by letters.

Figure 2 shows that the individual aLECs (Figures 2A,C) or smaller regions of the lens epithelium (Figures 2B,D) are damaged. There are several smaller lesions of this type that are present diffusely (Figures 2A,B). The damaged aLECs can be surrounded by normal aLECs (Figure 2C) or can be next to each other (Figure 2D). Single healthy and damaged aLECs are presented to clearly show the differences with the dents on the apical side centrally toward the nucleus on the damaged aLEC (Figure 2E) and not on the healthy aLEC (Figure 2F). Pathological aLECs do not only have an apical indent, but their surface is not smooth compared with the normal aLEC.

**Figure 3:**
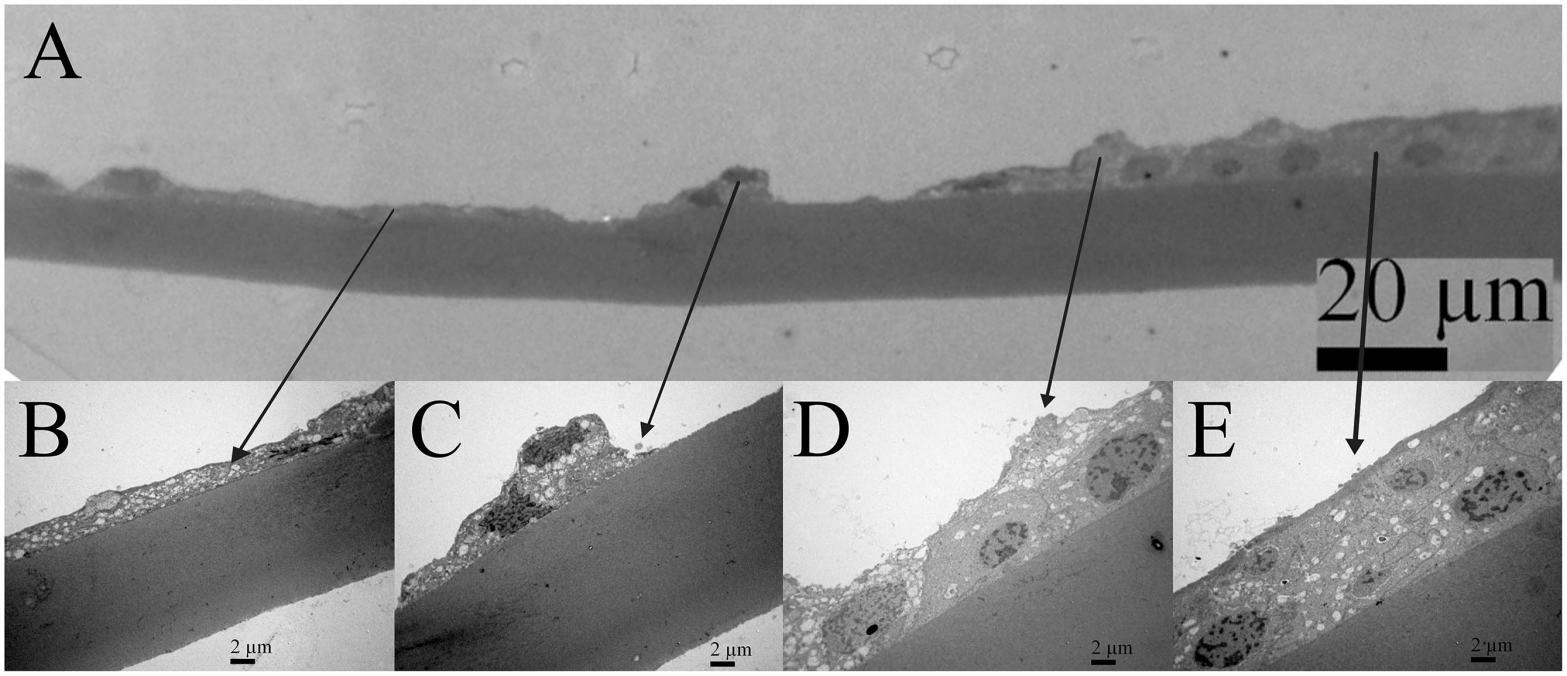
Transmission electron microscopy (TEM) of the 16 years old female juvenile cataract patient's aLC. TEM is showing thinning of the lens epithelium and degradation of the aLECs where the nucleus is degenerated **(A–C)** with the spacing between the adjacent aLECs laterally, in comparison with the non-thinned lens epithelium where aLECs nucleus is intact **(A,D,E)**.

Transmission electron microscopy composite of the same 16 years old female patient (Figure 3) is made from the four images of higher magnifications, and the same area at the lower magnification. The four images show the characteristic regions (Figures 3B–E), in which locations are visible on the low magnified image (Figure 3A). The changes in the lens epithelium are unevenly distributed: the thinning of the lens epithelium (Figures 3A,B), the degradation of the aLECs where the nucleus of the aLECs has degenerated with the spacing between the adjacent aLECs laterally due to the absent cells (Figures 3A,C), and the non-thinned lens epithelium (Figures 3A,D,E). The aLECs with the surface that is not smooth could be considered to have an apical indent (Figure 3D, left). The aLECs exhibit a great variety in “heights”, and create an irregular margin of the apical lens epithelium. In addition, multilayering of cells is visible (Figures 3A,E).

### Patient 2 (41 Years Old Female)

**Figure 4:**
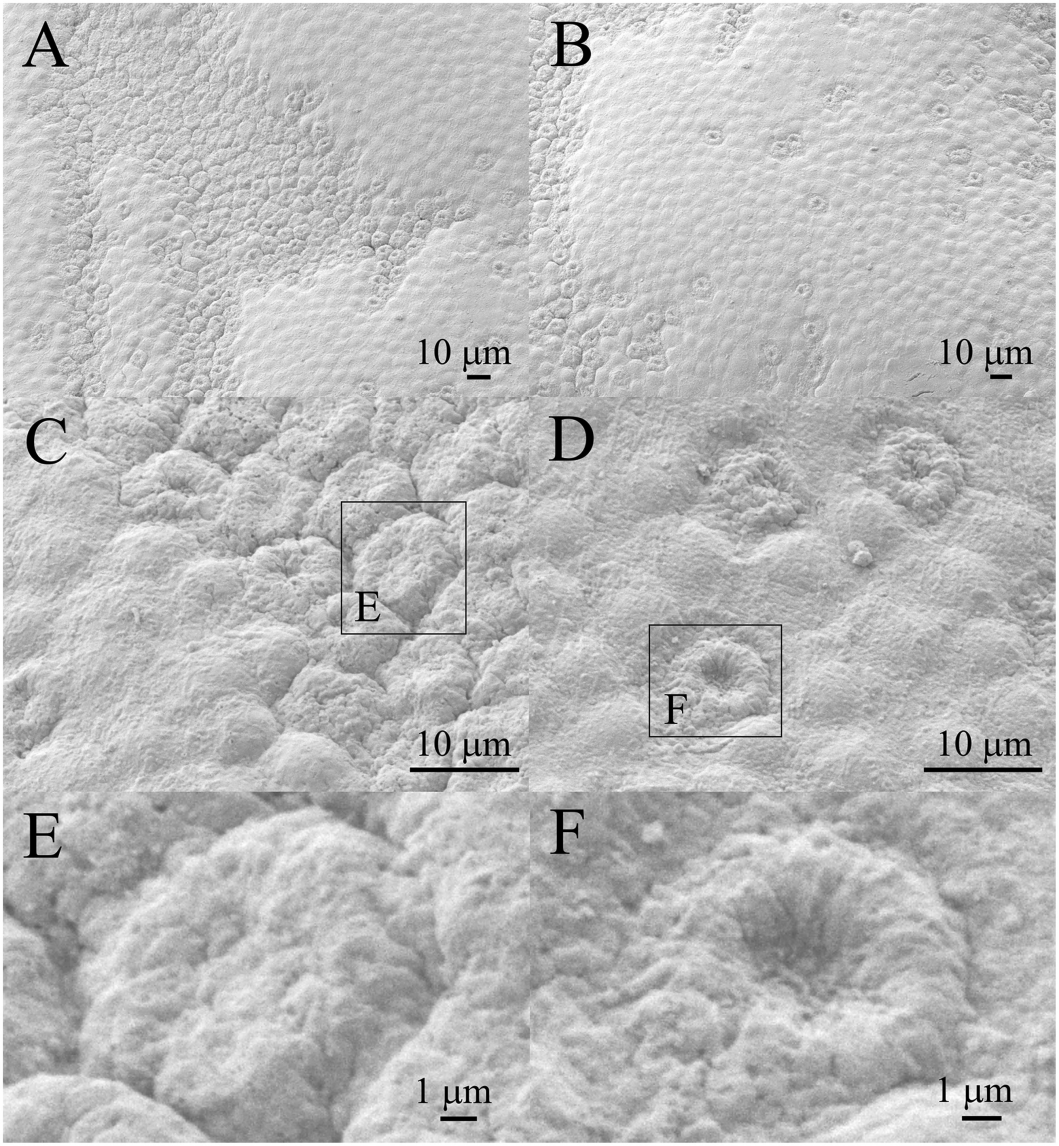
Scanning electron microscopy of the 41 years old female presenile cataract patient's with aLC. SEM is showing that both the larger regions of the lens epithelium **(A)** and the individual aLECs are damaged **(B)**, with the different degree of dents on the apical side centrally toward the nucleus on different damaged aLECs **(C–F)**. The squares represent the regions that are enlarged and are labeled by letters.

Figure 4A shows the lens epithelium region in which a bigger part of the aLECs is damaged, while Figure 4B shows the lens epithelium region in which only individual aLECs are damaged and the aLECs surrounding them are healthy. Several lesions of both types are present diffusely and are pronounced. Figure 4C shows two enlarged regions, left with the normal aLECs and right with the damaged aLECs. Figure 4D shows enlarged individual damaged aLECs surrounded by the normal aLECs. Different degrees of damage can be observed for different aLECs, as on some, the dents on the apical side centrally toward the nucleus are less pronounced (Figure 4E) and on the others are more pronounced (Figure 4F).

**Figure 5:**
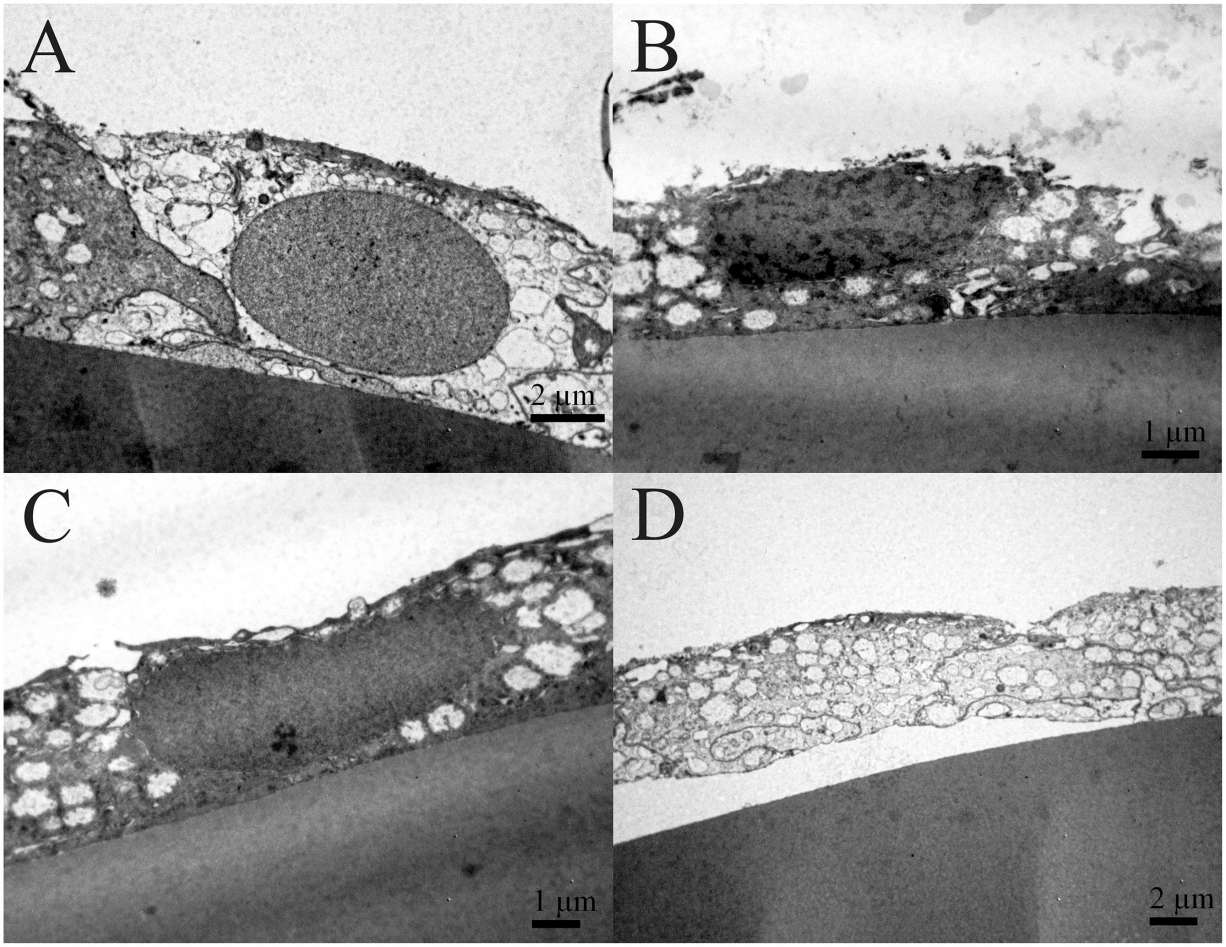
Transmission electron microscopy of the 41 years old female presenile cataract patient's aLC. TEM is showing the lens epithelial regions in which the cytoplasm of aLECs is vacuolated and thinned, the cells are segmentally concave, and the nuclei are compressed and elongated **(B,C)**. The shelling of the cells **(D)** in comparison with the normal aLEC **(A)** is also shown.

In Figure 5, the cross-section of the part of the lens capsule preparation of the same patient is considered which shows unevenness in thinning of the lens epithelium. The changes on the lens capsule are diffuse, but they do not look as pronounced as on SEM. While Figure 5A shows relatively normal cell, Figures 5B,C show the lens epithelial regions in which the cytoplasm of aLECs is vacuolated and thinned; the cells are segmentally concave and the nuclei are compressed and elongated. Transparent vacuoles of various sizes are visible. While nuclei look different, their shape can also reflect the thinner and flatter shape of cells; the concavity is subtle compared with that shown in the SEM. Figure 5D shows the shelling of the cells with the loose connection between the cell and the basement membrane, leading to complete detachment of the cell from the basement membrane.

### Patient 3 (29 Years Old Male)

**Figure 6:**
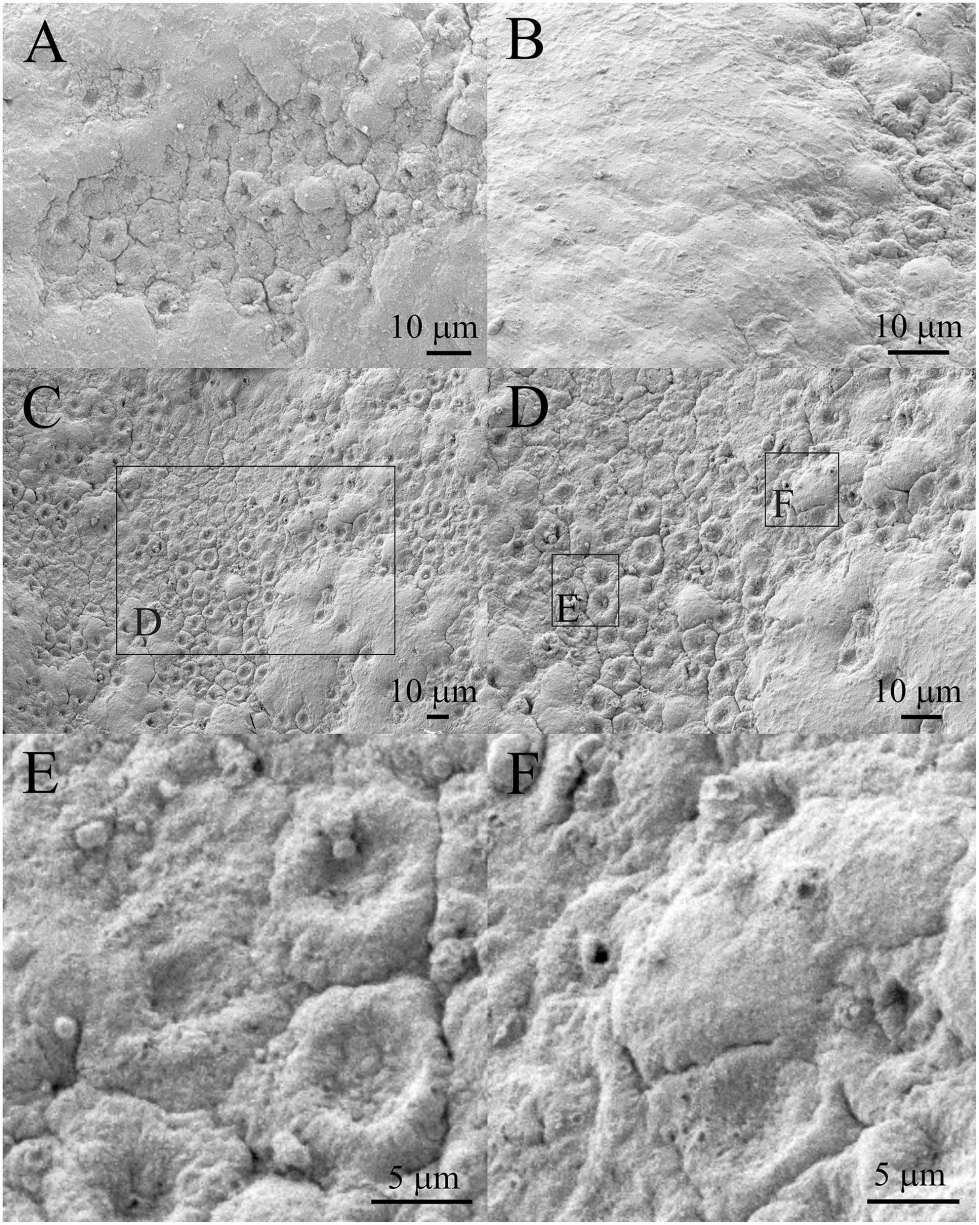
Scanning electron microscopy of the 29 years old male presenile cataract patient's aLC. SEM is showing the larger regions of lens epithelium that are damaged **(A–D)**. The small holes can be observed **(E,F)**. The squares represent the regions that are enlarged and are labeled by letters.

Figure 6A shows the region of damaged aLECs surrounded by the region of normal/healthy aLECs. Figure 6B shows the region of normal/healthy aLECs on the left and the smaller region of damaged aLECs on the right. Several lesions are present diffusely and are pronounced. Figure 6C shows the bigger region with damaged aLECs, which is enlarged (Figure 6D), so that the changes on individual aLECs can be shown better. Small holes (around 1 μm^2^ or less) can be seen on damaged aLECs (Figures 6E,F).

**Figure 7:**
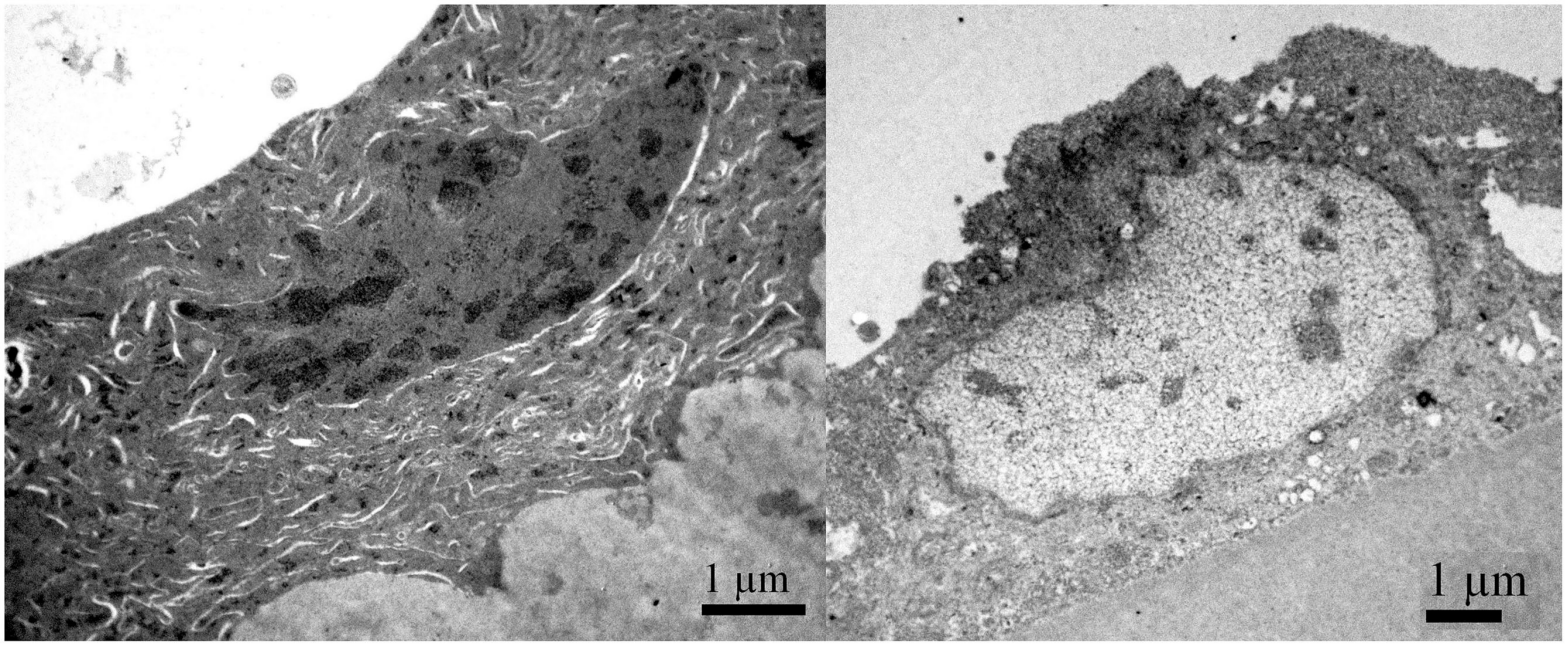
Transmission electron microscopy of the 29 years old male presenile cataract patient's aLC. TEM is showing that the cytoplasm of aLECs is thinned, the cells are segmentally concave, the nuclei are compressed and elongated, and the chromatin is condensed.

Figure 7 shows the TEM of the same patient. The cytoplasm of aLECs is thinned, the cells are segmentally concave. The degradation of the aLECs with the signs of necrosis is visible, invading the underlying lens capsule that has lost its smooth surface. The nuclei are compressed, elongated, and condensed.

### Patient 4 (39 Years Old Male)

**Figure 8:**
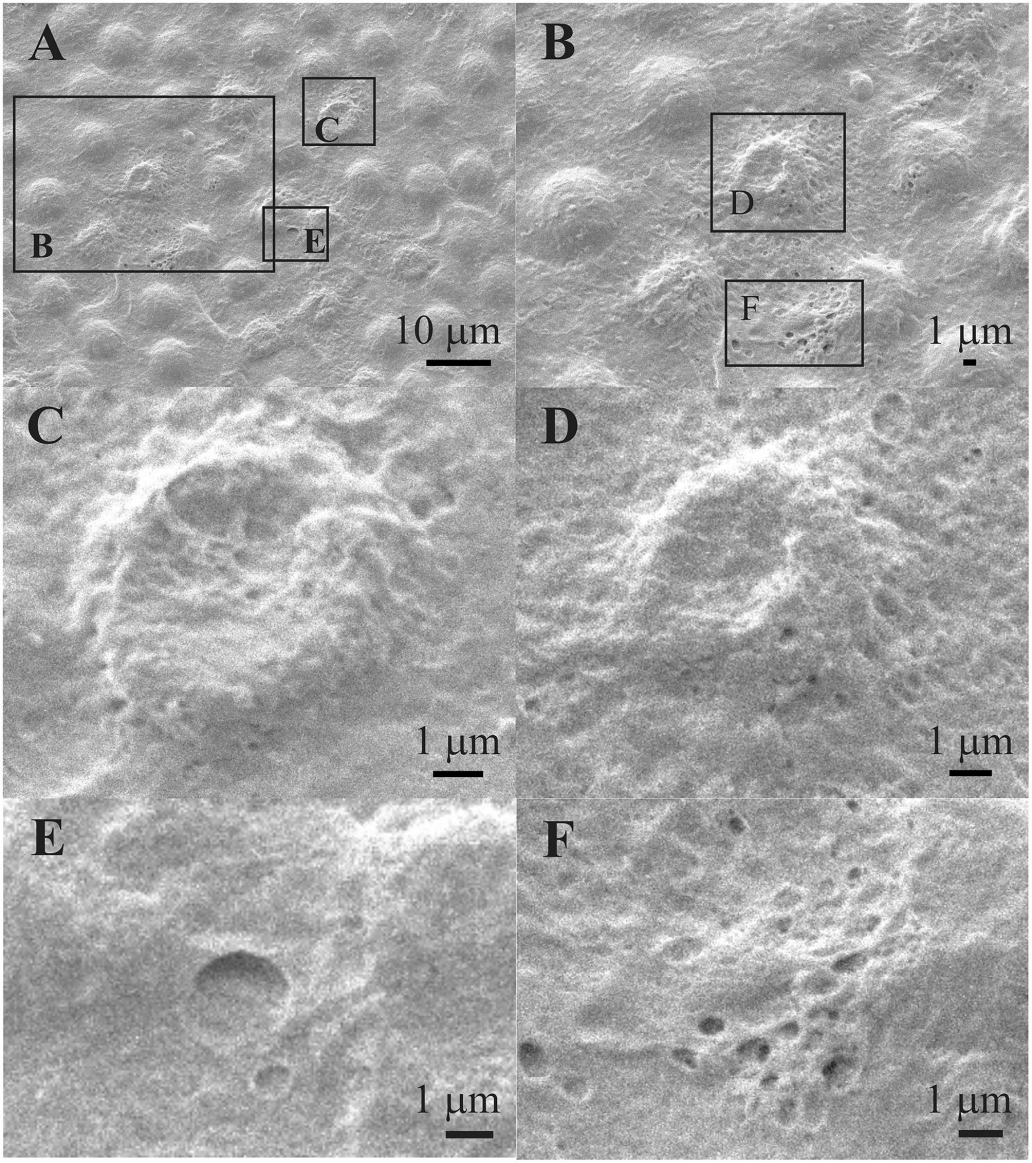
Scanning electron microscopy of the 39 years old male presenile cataract patient's aLC. SEM is showing damaged individual aLECs **(A,B)** with the different degree of dents on the apical side centrally toward the nucleus **(C,D)**. The small holes can be observed on the damaged lens epithelium **(E,F)**. The squares represent the regions that are enlarged and are labeled by letters.

Figure 8 shows that the individual aLECs are damaged. Figure 8B shows an enlarged region in the center of which is the damaged aLECs with the dents on the apical side centrally toward the nucleus. Figures 8C,D show enlarged individual damaged aLECs with different degrees of dents on the apical side centrally toward the nucleus. Small holes (around 1 μm^2^ or less) can be seen on the damaged region of the lens epithelium (Figures 8E,F).

**Figure 9:**
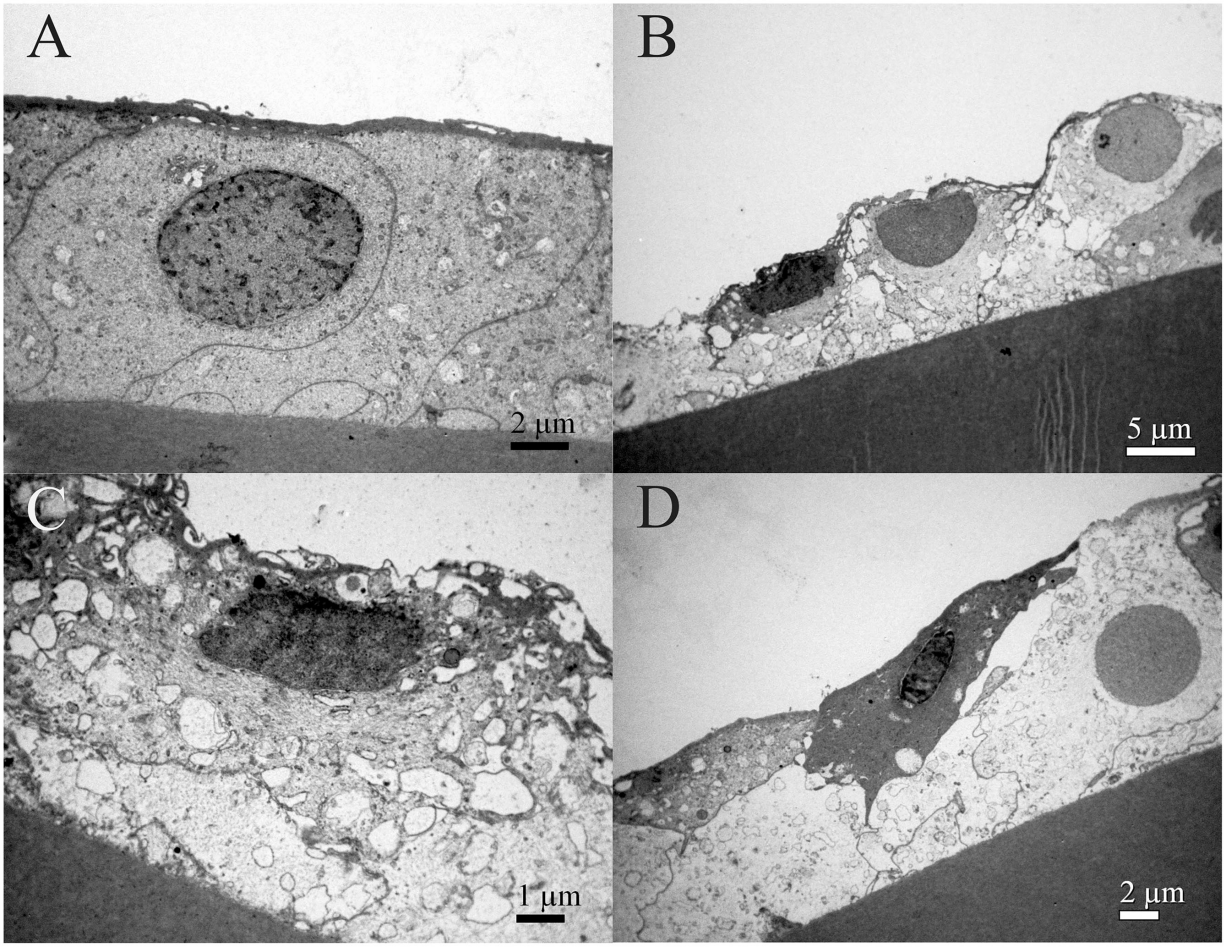
Transmission electron microscopy of the 39 years old male presenile cataract patient's aLC. TEM is showing thinning of the lens epithelium **(B)** and degradation of the aLECs where the nuclei are degenerated **(B–D)** and the cytoplasm of aLECs is vacuolated **(B, C)**, in comparison with the non-thinned lens epithelium where aLECs nucleus is intact **(A)**.

Figure 9 shows the TEM of the same patient. While Figure 9A shows a normal lens epithelial region where the aLECs nucleus is intact, Figures 9B–D shows the lens epithelial regions in which the thinning of the lens epithelium (Figure 9B) and degradation of the aLECs with the degenerated nuclei (Figures 9B–D) and the vacuolated cytoplasm of aLECs (Figures 9B,C) are visible. The loss of the regular shape of LECs and their nuclei is visible. Features of intracellular edema can be seen. A transparent vacuole can be observed between two cells making the loose connection of cells (Figure 9D).

### Patient 5 (45 Years Old Male)

**Figure 10:**
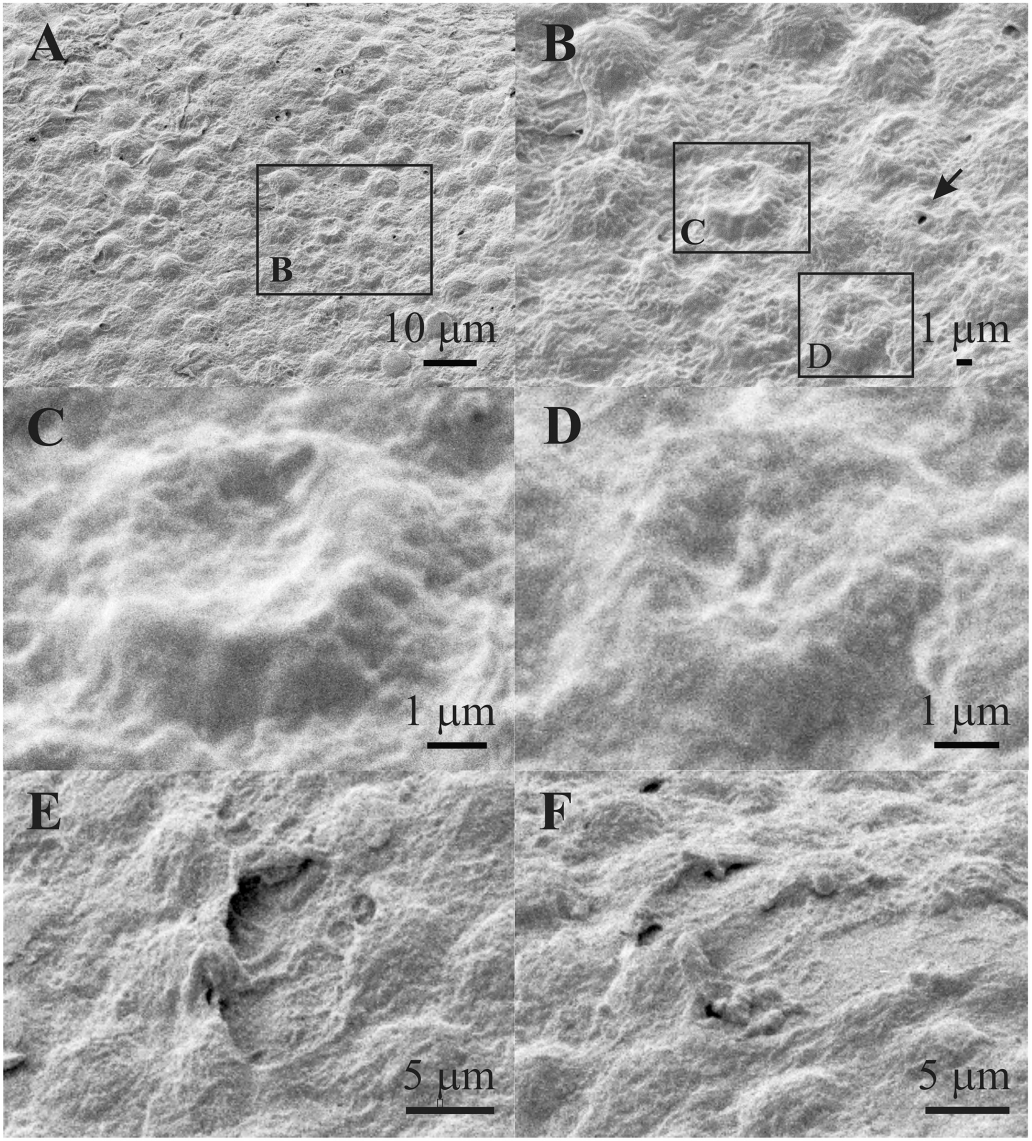
Scanning electron microscopy of the 45 years old male presenile cataract patient's aLC. SEM is showing that the smaller regions of the lens epithelium **(A,B)** are with the damaged aLECs having different degree of dents on the apical side centrally toward the nucleus **(C,D)**. Bigger holes can be seen on damaged region of the lens epithelium **(E,F)**. The squares represent the regions that are enlarged and are labeled by letters.

Figures 10A,B show that the smaller regions of the lens epithelium (Figures 10A,B) have the damaged aLECs with the dents on the apical side centrally toward the nucleus. The damaged aLECs can be next to each other (Figure 10B). Figures 10C,D show enlarged individual damaged aLECs with different degrees of dents on the apical side centrally toward the nucleus. Bigger holes (more than 5 μm^2^) can be seen on the damaged region of the lens epithelium (Figures 10E,F).

**Figure 11:**
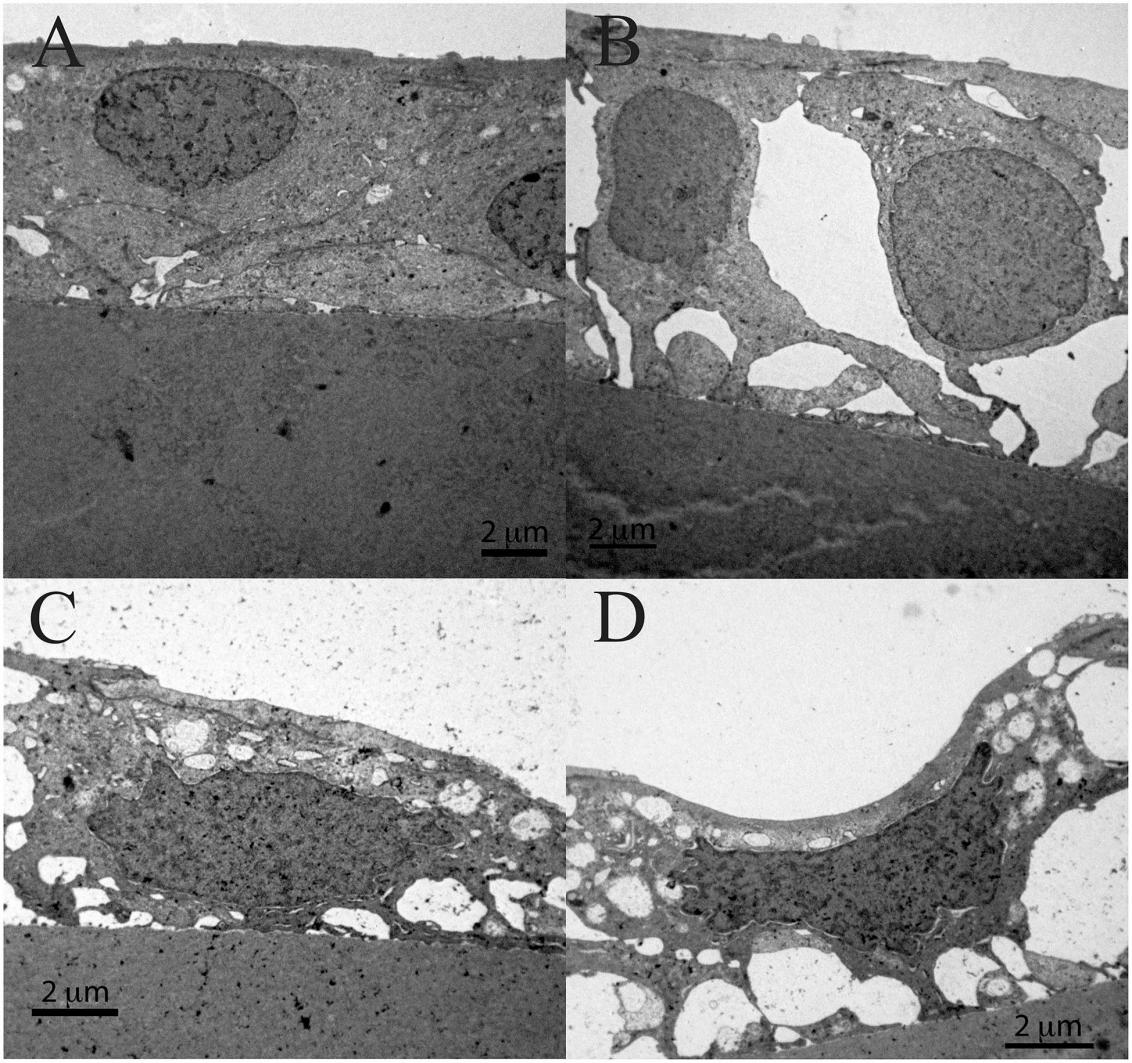
Transmission electron microscopy of the 45 years old male presenile cataract patient's aLC. TEM is showing the lens epithelial regions in which the large intercellular space is present **(B)**, the cytoplasm of aLECs is vacuolated and thinned, and the nuclei are compressed and elongated **(C,D)** and importantly, the cell is segmentally concave **(D)**, in comparison with the non-thinned lens epithelium where aLECs nucleus is intact **(A)**.

Figure 11 shows the TEM of the same patient. While the non-thinned lens epithelium where the aLECs nucleus is intact is shown in Figure 11A. Figures 11B–D show the lens epithelial regions in which large intercellular space is present (Figure 11B), the cytoplasm of aLECs is vacuolated and thinned, and the nuclei are compressed and elongated (Figures 11C,D). More importantly, the cell is segmentally concave (Figure 11D), so the loss of the regular shape of aLECs and their nuclei is visible. Transparent vacuoles of various sizes can be observed between the cells and between the cells and the basic membrane making loose connection with the basement membrane (Figures 11B–D).

## Discussion

We have shown by using SEM and TEM that the aLECs of anterior lens capsule in idiopathic presenile cortical cataract have pronounced abnormal structural features reflected in the form of thinning of the lens epithelium, with segmentally concave aLECs, the dents on the aLECs apical side centrally toward the nucleus, and compressed and elongated nuclei. In some regions, the single aLECs in lens epithelium are damaged, while in the others, larger zones of the lens epithelium are damaged. So, there are the regions with mild lens epithelial impairment, and the regions with larger epithelial impairment. To the best of our knowledge, this is the first time that the structural features as the dents, the selective concavity of some LECs, at their apical side centrally toward the nucleus, are shown on the lens epithelium of patients with presenile cataract when studied simultaneously by SEM and TEM in the same preparation.

Interestingly, when compared with the lens epithelia from 49 patients with different senile cataract types (nuclear (N), cortical (C), N+C, intumescent white cataract, and retinitis pigmentosa) that we studied by SEM and TEM, we found similar changes of the aLECs structure in the form of the dents only in 2 patients (4%), while in other 47 patients (96%) no such changes were observed (10, 11). This suggests that the damaged epithelial regions are due to the presenile cataract and are not an artifact of the surgical extraction and separation from the normal contact with fiber cells or tissue processing.

Already with clinical examination before the cataract surgery, the subcapsular changes in the opacity are visible both on the anterior part of the lens and in the posterior subcapsular part. As shown in high resolution OCT images, vacuolation can be seen between the lens capsule and cortex, probably representing water accumulation under the capsule (Figure 1), which may leak further along the lens fibers to the posterior pole causing posterior subcapsular cataract. Generally, in any damage of the lens epithelium and its transport function, water passively enters the lens. Lens epithelium plays a key role in maintaining the levels of electrolytes and water in the lens, which are necessary for lens transparency (6, 12, 13). Therefore, an influx of the water through the impaired lens epithelium may represent the mechanism of development of the presenile cataract and may be indicative of a common mechanism of cataract development, involving the water mechanism. Laursen and Fledelius in 1979 (14) have already suggested that the opacities of the anterior capsular/subcapsular layers and, in particular, a posterior subcapsular cataract, may be associated with an increase in permeability of the lens “membrane” consisting of the basement membrane + the epithelium anteriorly and the lens basement membrane posteriorly.

In all five patients, regions of degenerated aLECs were found. The death of aLECs leads to rearrangement of aLECs, which may further lead to uncoupling of cells, which is vital for the maintenance of the transparency of lens (15). It was shown that such uncoupling of cells and the breakdown of aLECs intercellular connectivity causes dysfunction of active transport of electrolytes, causing passive inward movement of water and the progression of cortical cataract development (16).

Lens epithelial cell apoptosis was suggested to be a common cellular basis for non-congenital cataract development in humans and animals (17). Depletion of patches of aLECs eliminates homeostatic epithelial cell control of the underlying fiber cells, leading to the impairment of the integrity and transparency of these underlying fiber cells (18). By SEM and TEM, we found the regions with the impaired lens epithelium, where the aLECs are damaged, often with the concave nuclear region.

Anterior lens epithelium in presenile cataract was observed by SEM and TEM only in one study (19) in which the abnormal structural features of aLECs were observed in the presenile compared with age-related cataract patients. While TEM and SEM showed some common changes as also observed in our presenile cataract samples, such as vacuolated cytoplasm, and elongated nuclei, holes formed by the aLECs stretching were only seen in the patients with presenile cataract. In addition to these, we show features not described previously, in particular, the dents at aLECs apical side centrally toward the nucleus which were very well distinguished by SEM. The swollen cells and spheres were observed in intumescent cataract lens epithelia (10).

In comparing the cellular features in presenile cataracts to those already described in senile cataracts, it is important to depict specific morphological features that would only be inherent to presenile cataracts. However, there are very few recent research that studied SEM and TEM features of the lens epithelium in senile cataracts and they do not distinguish cortical from nuclear senile cataracts, whereas cortical cataracts would be more relevant to compare. These document irregular apical surfaces of the lens epithelium (20–22). TEM examination revealed ultrastructural abnormalities, such as transparent vacuoles of various sizes that were detected between the cells and between cells and the basal membrane, influencing the appearance of both the nucleus and the whole cell, and were detected in all patients with age-related cataracts. Additionally, diffuse intracellular edema was observed, and was more extended and more frequently observed in the exfoliation syndrome group. Many other ultrastructural features were shown in all patients with age-related cataracts. The irregularly shaped nuclei and aLECs were also observed. Cells exhibited a great variety in “heights”. The cells were loosely connected among them and with the basement membrane or were in some cases absent. Sometimes the epithelium was completely detached from the basement membrane. Very often there was more than one layer of cells (21). However, in exfoliation syndrome, SEM did not show the dents on the aLECs as described in our studies (20). Large intercellular and a few intracellular vacuoles were also seen in the anterior part of the epithelium, both light- and electron-microscopically in senile cataract lenses (23). Similar degenerative changes in aLECs were shown in different types of cataracts, such as multilayered cells, nuclei of abnormal diameters and shapes, vacuolation of nuclei, and cytoplasm (24). These studies report many similar alterations to the ones observed in our study by TEM, even though none of the patients had presenile cataracts. There are certainly many common features, however, we report others, previously not described, especially by SEM, as the dents, the selective concavity of some LECs, at their apical side centrally toward the nucleus. Although it shares similarities to senile cataracts, the occurrence of such changes in such young people is unusual, so it is worth studying.

A limitation of this study is the unavailability of the normal lens capsules for comparison, as we obtain lens epithelium after cataract surgery and the changes were compared with common nuclear cataract, in which lens epithelia were largely normal (10). Another limitation of the study was the small sample size as such cataracts are relatively rare. For this reason, these findings may not be regarded as the general principle but as a report of changes documented by both, SEM and TEM, along with the high-resolution preoperative OCT in the same preparation not previously shown in this type of cataract, as a contribution toward finding the specific origins of dysfunction of the lens epithelium in this type of cataract.

In conclusion, structural studies of the presenile cataract lens epithelia by SEM and TEM show abnormal distinguishable features present in presenile idiopathic cortical cataract that may play a role in water accumulation and cataract formation.

## Data Availability Statement

The raw data supporting the conclusions of this article will be made available by the authors, without undue reservation.

## Ethics Statement

The studies involving human participants were reviewed and approved by the National Medical Ethics Committee of the Republic of Slovenia and all patients signed informed consent before the operation. Written informed consent to participate in this study was provided by the participants' legal guardian/next of kin.

## Author Contributions

MH: conceptualization, funding acquisition, resources, and supervision. SA, KD, and AH: data curation, investigation, and validation. SA: formal analysis, visualization, and writing—original draft. KD and AH: methodology. SA and MH: project administration and writing—reviewing and editing. All authors contributed to the article and approved the submitted version.

## Funding

This research was funded by the Slovenian Research Agency (ARRS), Ljubljana, Slovenia, program P3-0333.

## Conflict of Interest

The authors declare that the research was conducted in the absence of any commercial or financial relationships that could be construed as a potential conflict of interest.

## Publisher's Note

All claims expressed in this article are solely those of the authors and do not necessarily represent those of their affiliated organizations, or those of the publisher, the editors and the reviewers. Any product that may be evaluated in this article, or claim that may be made by its manufacturer, is not guaranteed or endorsed by the publisher.

## Supplementary Material

Figure S1 Clinical imaging of 39 years old male presenile cataract patient's lens before cataract surgery. Spectralis OCT **(A)** and the slit lamp **(B,C)** images are shown. Subcapsular opacities and vacuoles between the lens capsule and cortex can be seen.

Figure S2 Clinical imaging of 45 years old male presenile cataract patient's lens before cataract surgery. Spectralis OCT **(A)** and the slit lamp **(B,C)** images are shown. Vacuoles and subcapsular opacities between the lens capsule and cortex are visible.
