# Supplementary figures and images for: Structural Characteristics of the Lens in Presenile Cataract
Source: Front Med (Lausanne). 2021 Dec 22;8:802275. doi: 10.3389/fmed.2021.802275 (PMC8727346; doi:10.3389/fmed.2021.802275)

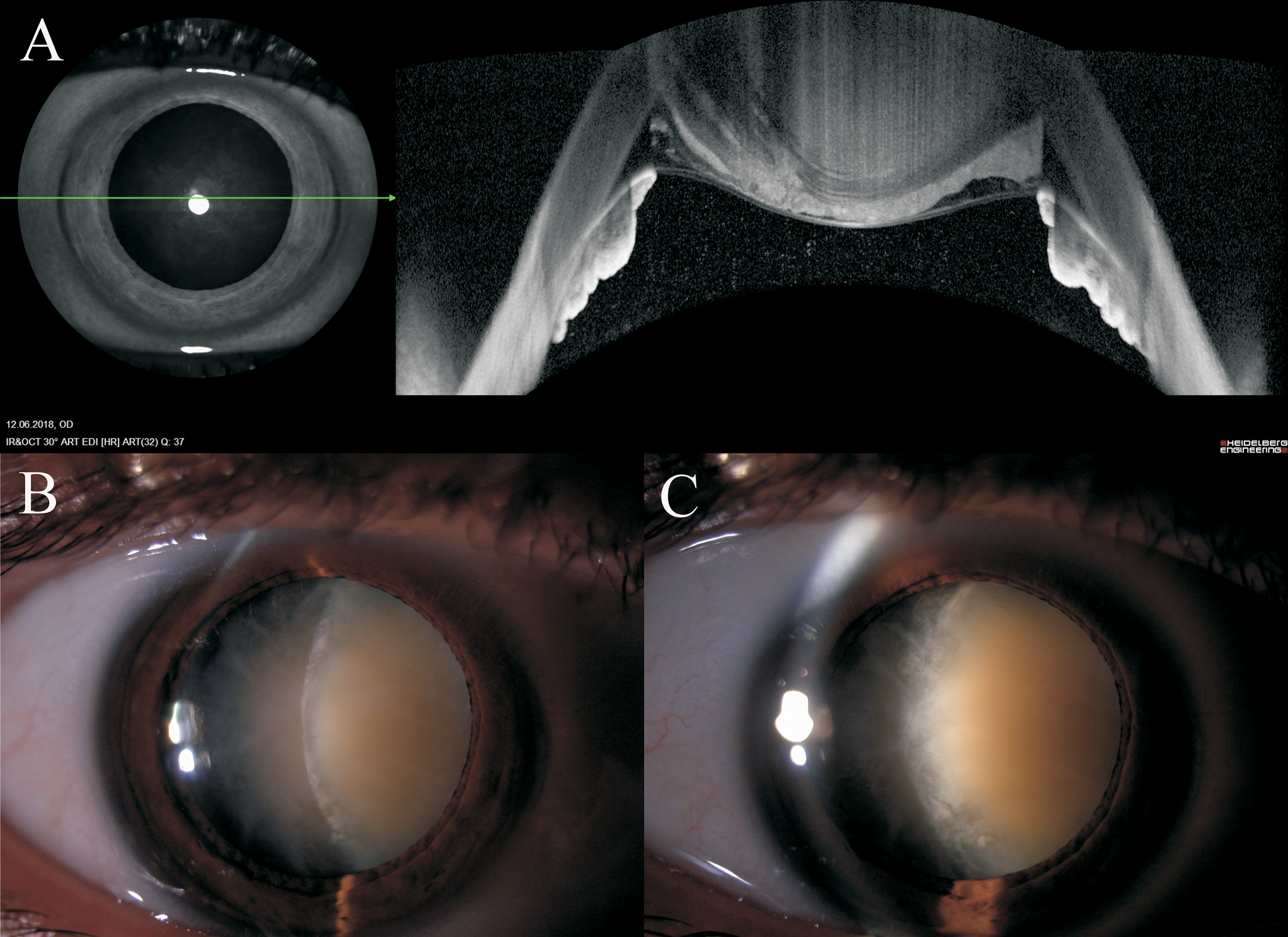

Supplement: Figure S1 — Clinical imaging of 39 years old male presenile cataract patient's lens before cataract surgery. Spectralis OCT (A) and the slit lamp (B,C) images are shown. Subcapsular opacities and vacuoles between the lens capsule and cortex can be seen. [file Image_1.TIF]

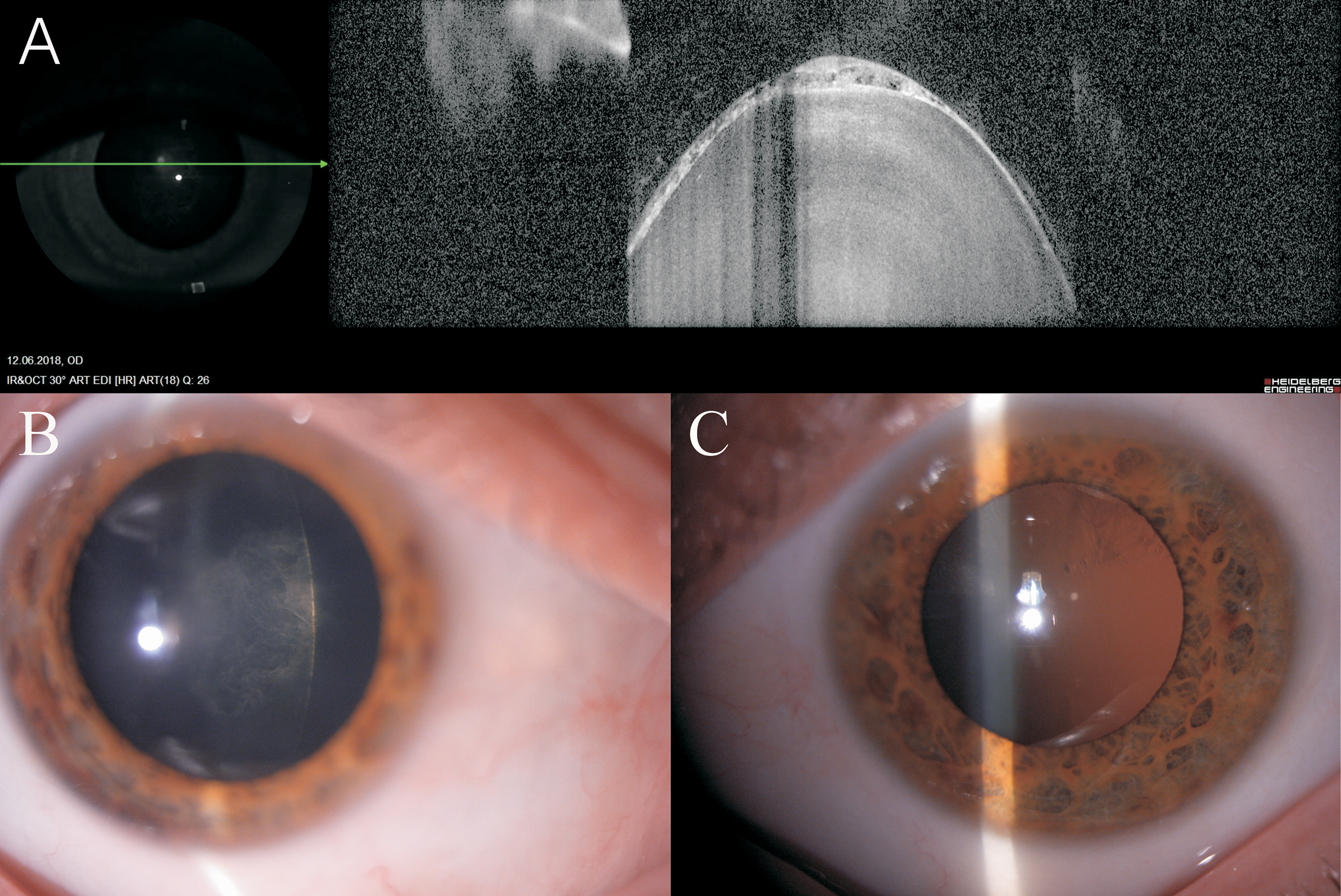

Supplement: Figure S2 — Clinical imaging of 45 years old male presenile cataract patient's lens before cataract surgery. Spectralis OCT (A) and the slit lamp (B,C) images are shown. Vacuoles and subcapsular opacities between the lens capsule and cortex are visible. [file Image_2.TIF]
